# 

**Published:** 1898-08

**Authors:** 


					﻿ESTABLISHED IS55.	SUBSCRIPTION, H.00.
ATLANTA
MEDIGflli OD SU Wftfe
1	' SURGEON GENERAL’S OFFICE
__________JOURNAL. AUG.-G—18^8 I
newUserhss. Atlanta, Ga., August, 1898. No. 6.
				

